# Role of Acclimatization in Weather-Related Human Mortality During the Transition Seasons of Autumn and Spring in a Thermally Extreme Mid-Latitude Continental Climate

**DOI:** 10.3390/ijerph121214962

**Published:** 2015-11-26

**Authors:** Christopher R. de Freitas, Elena A. Grigorieva

**Affiliations:** 1 School of Environment, University of Auckland, Auckland 1142, New Zealand; 2 Institute for Complex Analysis of Regional Problems, Far Eastern Branch, Russian Academy of Sciences, Birobidzhan 679016, Russia; eagrigor@yandex.ru

**Keywords:** acclimatization, heat strain, cold strain, human bioclimate, Acclimatization Thermal Strain Index, physiological adaptation, seasonal mortality

## Abstract

Human mortality is closely related to natural climate-determined levels of thermal environmental stress and the resulting thermophysiological strain. Most climate-mortality research has focused on seasonal extremes during winter and summer when mortality is the highest, while relatively little attention has been paid to mortality during the transitional seasons of autumn and spring. The body acclimatizes to heat in the summer and cold in winter and readjusts through acclimatization during the transitions between the two during which time the body experiences the thermophysiological strain of readjustment. To better understand the influences of weather on mortality through the acclimatization process, the aim here is to examine the periods that link very cold and very warms seasons. The study uses the Acclimatization Thermal Strain Index (ATSI), which is a comparative measure of short-term thermophysiological impact on the body. ATSI centers on heat exchange with the body’s core via the respiratory system, which cannot be protected. The analysis is based on data for a major city in the climatic region of the Russian Far East characterized by very hot summers and extremely cold winters. The results show that although mortality peaks in winter (January) and is at its lowest in summer (August), there is not a smooth rise through autumn nor a smooth decline through spring. A secondary peak occurs in autumn (October) with a smaller jump in May. This suggests the acclimatization from warm-to-cold produces more thermophysiological strain than the transition from cold-to-warm. The study shows that ATSI is a useful metric for quantifying the extent to which biophysical adaptation plays a role in increased strain on the body during re-acclimatization and for this reason is a more appropriate climatic indictor than air temperature alone. The work gives useful bioclimatic information on risks involved in transitional seasons in regions characterized by climatic extremes. This could be handy in planning and managing health services to the public and measures that might be used to help mitigate impacts.

## 1. Introduction

An increase in mortality associated with heat and cold has been reported for a variety of diseases, among them, respiratory disease, cardiovascular disease, nervous systems disorders, and kidney and urinary system diseases, and likely biological mechanisms that cause them have been identified [1,2,3,4,5,6]. It has also been found that these effects are most pronounced among the elderly. Since thermal environmental conditions can have a deadly effect on the health of individuals who are already ill from other causes, often the other causes are given as the reason for death [7]. Moreover, thermal conditions may affect individuals differently depending on the disease. Because they are difficult to diagnose with certainty, these deaths can be misclassified as due to causes other than thermal [7]. For all these reasons, all-cause non-accidental mortality may be a better indicator of the extent to which human mortality is closely related to levels of thermal environmental stress and resulting thermophysiological strain.

Most all-cause non-accidental mortality-climate research to date has focused on seasonal extremes when mortality is the highest (winter) or most affected by extreme heat events (summer), and it is well established that the relationship differs in each of these two seasons [8,9,10,11,12,13,14,15,16,17,18], but relatively little research has considered patterns of mortality during the transitional seasons of autumn and spring, periods when temporal autocorrelations in mortality records suggest the existence of important shoulder season connections [19]. The body acclimatizes to heat in summer and cold in winter. The readjustment through reacclimatization occurs during the transitions between the two. Consequently, the thermophysiological strain of readjustment may be expected to occur in the changeover seasons of spring and autumn. This seasonal adaptation is especially important for regions with climates characterized by very hot summers and extremely cold winters and big temperature changes between these main seasons through the transition periods. In contrast to the effects of absolute thermal environmental conditions, the effects of acclimatization have not been thoroughly investigated, although it is known that large day-to-day temperature changes may affect the respiratory system of humans, and especially children [20,21,22]. To better understand the influences of weather on mortality through the acclimatization process, the aim here is to examine the periods that link summer and winter.

### Acclimatization

The human body is, as a rule, thermophysiologically well adapted to its home climate. A change of setting due, say, to travel between places where climatic conditions are quite dissimilar, can have a physiological cost. When differences are large, there is a period of short-term acclimatization adjustment during which the body might experience additional thermally induced physiological strain [23,24,25,26,27,28,29,30,31,32,33]. This adaptation period is not risk free, so it may be necessary to take into account the impact of the change in physiological well-being during the period of adjustment.

Acclimatization is a natural process of gradual physiological adjustment of the human body as it gets used to new climatic conditions. It is the ability of the human body to undergo physiological adaptations so that the stress of a new climatic environment is ultimately less severe. The primary physiological adaptations to heat acclimatization include: an earlier onset and higher rate of skin blood flow; an increased sweat rate with earlier onset and more dilute concentration of sweat; decreased electrolyte loss and greater resistance to dehydration; a decrease in basal metabolic rate and heart rate; a decrease in perceived exertion; and a decrease in oxygen consumption at a given activity level or metabolic rate [34,35,36,37]. It is noteworthy heat acclimatization does not allow higher storage of heat in human tissues [38,39].

The physiological adaptations to cold acclimatization are thought to be small and depend on the severity and duration of exposure [40], but according to experiments by Lazar *et al.* [31], cold acclimatization resulted in elevated resting metabolism, a reduced fall in body temperature during acute cold stress, reduction in shivering, improvement in cold induced vasodilation and thermoregulatory efficiency and less of a rise in blood pressure and heart rate. In an unacclimatizated body, absence of the above adaptations adds to the thermophysiological strain experienced under new climatic conditions. A more detailed discussion of the physiological mechanisms that account for differences in acclimatization processes in the transition from cold to warm and from warm to cold environments are given in [41,42].

Acclimatization thermal loading can be used in an index (ATSI) to quantify the thermophysiological impact of the changes due to a lack of acclimatization; and applied to people moving between contrasting climatic regions [41,42]. The ATSI scheme is based mainly on Russian research, but the extent to which the index validation of ATSI against empirical data is not clear. In general terms, there are two ways to test ATSI against empirical data. The first is by way of laboratory experimentation, but there are major logistical and practical problems such as the need for specialized equipment and clinical facilities that are in the realm of the physiologist. The second method is to statistically compare climate data with physiological data, such as to test ATSI against health impacts of heat waves and cold snaps, or impacts during transitional seasons in extreme climate regimes. The latter approach is the focus here.

## 2. Method

The study uses the Acclimatization Thermal Strain Index (ATSI), which is a relative measure of short-term thermophysiological impact on the body. The analysis is based on data for Khabarovsk, a major city in the climatic region of the Russian Far East characterized by very hot summers and extremely cold winters. Determination of ATSI is based on the earlier work of De Freitas and Grigorieva [41,42] summarized below.

ATSI centers on heat exchange with the body’s core via the respiratory system, which cannot be protected. Unlike other widely used bioclimatic indices, ATSI is a relative measure, based on physiological responses known to negatively impact human wellbeing. The processes involved centers on the regulation of heat exchange that is one of the fundamental ways the human body maintains a constant core temperature. Heat loss from the core immediately translates into physiological heat strain on the body, the first signs of which show up in heat exchange through the respiratory organs [32]. De Freitas and Grigorieva [41] show that the physiological significance of adjustment to changed thermal conditions may be expressed as an adjustment loading, or acclimatization thermal loading (ATL) and used ATL to quantify the physiological impact of the change expressed as an ATSI value.

The respiratory heat exchange between the lungs and the outside air (*Q_r_*) takes place by convection and involves both a dry heat flux and evaporative (latent) heat flux [43,44,45,46,47]. *Q_r_* can be quantified as:

*Q_r_ = P_l_ + LE* (1)

*P_l_ =* 2 × 10^−5^*w·b (T_l_ − T)* (2)

*LE =* 2.9 × 10^−2^*w (l_l_−l)* (3)

where *Q_r_* (W) is heat loss via respiratory organs, *P_l_* (W) is sensible (dry) heat loss from the respiratory tract caused by heating of inhaled air, *LE* (W) is evaporative heat loss caused from moisture loss from a surface of respiratory organs, *w* (l min^−1^) is the volume of inhaled air, *T* (°C) is temperature of inhaled (*i.e*., ambient) air, *T_l_* is temperature of exhaled air set at 35 °C; *l* (hPa) is vapor pressure of inhaled (*i.e*., ambient) air, *l_l_* is vapor pressure of exhaled air set at 56.3 hPa, and *b* (hPa) is atmospheric pressure taken to be 1000 hPa [41]. The coefficients (2 × 10^−5^) and (2.9 × 10^−2^) are for terms P*_l_* and *LE*, respectively, for the particular metabolic rate used here.

The human condition is that for a person standing relaxed with a metabolic rate of 90 W·m^−2^ and a 1.5 m^2^ body area. At the given metabolic rate, the volume of inhaled hair is 8 L·min^−1^. The respiratory heat exchange given by *Q_r_* occurs directly via ambient air entering into the body’s core through the respiratory tract. Because of this, the process simulated is independent of clothing, solar heat load, or any general heat flow considerations to or from the body surface.

ATSI describes the additional thermal loading on respiratory organs until full acclimatization is achieved. ATSI is defined as the ratio of the difference between respiratory heat losses for the acclimatized and non-acclimatized individual, expressed as a percentage as follows:

ATSI = 100 (*Q_rh_ − Q_r_’)* / *Q_rh_* (4)

where *Q_rh_* (W) is heat loss from respiratory organs for the acclimatized individual, and *Q_r_’* (W) is heat loss at for the new or unacclimatized state. An ATSI value of zero marks the transition ATL for unacclimatized individuals and represents the least stressful conditions. ATSI values less than zero indicate ATL due to lack of acclimatization to cold conditions, with increasing severity as the values become progressively more negative. Rising positive values of ATSI above zero indicate the onset of ATL due to lack of acclimatization to hot conditions.

In the current work ATSI is calculated for daily and monthly comparisons given by:

ATSI = 100 (*Q*_(*i*__− 1)_ − *Q_i_*) / *Q*_(*i*__− 1)_ (5)

where *i* is month, (*i* − 1) is previous month. For example, February is compared with January, and so on:

ATSI = 100 (*Q*_Aug_ − *Q_ii_*) / *Q*_Aug_ (6)

where *ii* is an autumn day, *Q*_Aug_ is respiratory heat loss in August, the last month of summer prior to the onset of autumn weather conditions. Equation (4) is the general expression relating *Q_rh_* and *Q_r_’*. In Equation (5), respiratory heat loss in months (*i* − 1), or in August in Equation (6), replaces *Q_rh_* for the acclimatized individual in Equation (4); likewise, respiratory heat loss in month *i* in Equation (5) and *ii* in an autumn day in Equation (6) is heat loss for the new or unacclimatized state *Q_r_’* in Equation (4).

### 2.1. Data

The weather and climate data (http://meteo.infospace.ru/) used are for the period from the start of 2000 through to the end of 2012. Three-hourly air temperature and vapor pressure data at 0000 h, 0300 h, 0600 h, 0900 h 1200 h, 1500 h, 1800 h and 2100 h are used in the initial computation and daily means generated from these.

The dataset necessary for an evaluation of seasonal mortality was sourced from the City Administration of Khabarovsk. It includes daily general mortality counts that span the period from the start of 2000 through to the end of 2012 sorted by gender and age. As of 1 January 2013, Khabarovsk had a population of 593,636 [48]. Age distributions above and below 65 years were identified to allow for separate assessment given that those over 65 are often more vulnerable to weather changes [1,3,4,49].

Often it is necessary to standardize raw mortality data to permit a proper spatial evaluation across geographic domains. This is because raw mortality totals are highly dependent on the age structure of a given location. An urban statistical area with an older population will have consistently higher death rates regardless of other factors; thus, direct comparisons of raw mortality totals cannot be made between cities with varying age structures, or within a city over a long period of time [12]. In present research we have a short period of 13 years and no comparison with other cities, so there is no need to standardize mortality.

### 2.2. Seasonal Mortality

An objective of the current work is creation of a seasonal mortality distribution. Mean daily mortality rates across the period of record are calculated and plotted. For example, for 1 January, assuming 13 years of mortality data, the mean for the 13 daily 1 January death rates are determined. This is repeated for every day of the year, creating 365 daily mortality counts, and a mean for the whole period. These daily data are than averaged for each month of the year and an Index of Seasonality (IS) for mortality derived as:

IS = (M*_i_* / M*_m_*)·100 (7)

where M*_i_* is mean mortality for a given month and M*_m_* is mean mortality for a month for the whole study period.

### 2.3. Mortality and Seasonal Acclimatization

Raw mortality data for the autumn season starting 1 September to 30 November were smoothed using a 15-day running mean. The 15-day time slot is chosen to balance the combined needs of: (a) smoothing fluctuations due to weather and other factors and (b) comparing mortality only within similar times of the year. On the one hand, averaging over as long a period as possible creates a smoother mortality curve. Conversely, averaging over a long period could lead to the merging of days that are different than the particular day in question due to seasonal differences in mortality. A compromise approach is to take one week on either side of the target day (15 days). A sensitivity analysis conducted by Kalkstein [50] showed that mortality curves created using a 15-day running mean did not differ substantially from those created using an 11 or 19-day smoothing procedure.

## 3. Results

**Figure 1:**
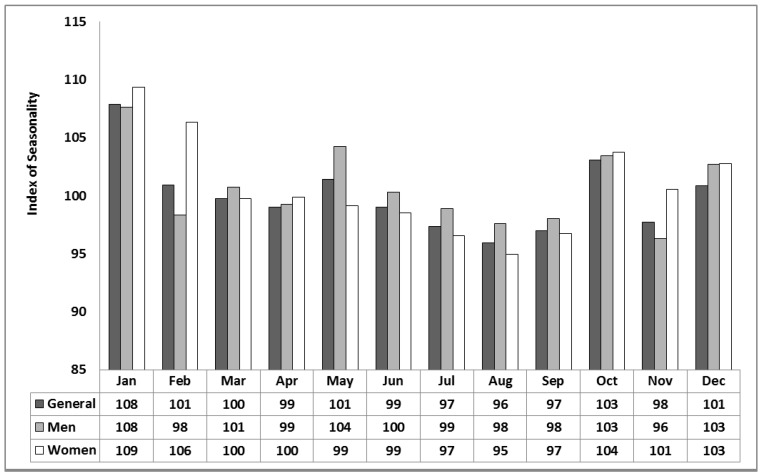
Monthly Index of Seasonality (IS) for Khabarovsk for the period 2000–2012 based on mean daily all-cause mortality for men, women and both men and women for all age groups.

**Figure 2:**
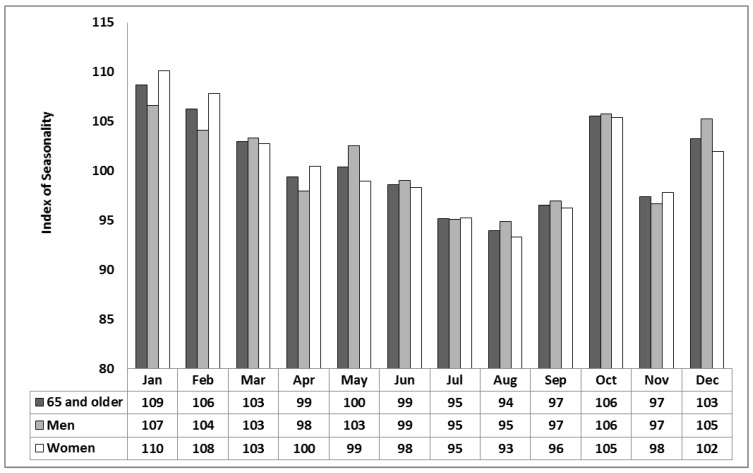
Monthly Index of Seasonality (IS) for Khabarovsk for the period 2000–2012 based on mean daily all-cause mortality for men, women and both men and women 65 years and older.

For the period 2000 to 2012 in Khabarovsk, females and males make up 44% and 56% of all-cause mortality, respectively, while deaths for those over 65 comprise 51% of the total. Using the IS metric defined in Equation (7), monthly mortality distributions for the study period are shown in Figure 1 and Figure 2. The results show mortality to be the highest in winter and the lowest in summer for males and females (Figure 1) and, in particular, those over 65 years of age (Figure 2). Peaks also show up in the transition seasons of spring and autumn, in particular, May and October. The strongest signal is apparent in October (Figure 1) for both males and females and stronger still those over 65 year of age (Figure 2). Based upon an unpaired, two-sample (2-tailed) *t*-test (alpha = 0.05), IS in October differs significantly than that in the other autumn months of September and November. The results show that IS captures a noticeable rise in mortality associated with acclimatization strain, especially from the hot to cold transition in autumn shown by a noticeable October spike in deaths.

**Figure 3:**
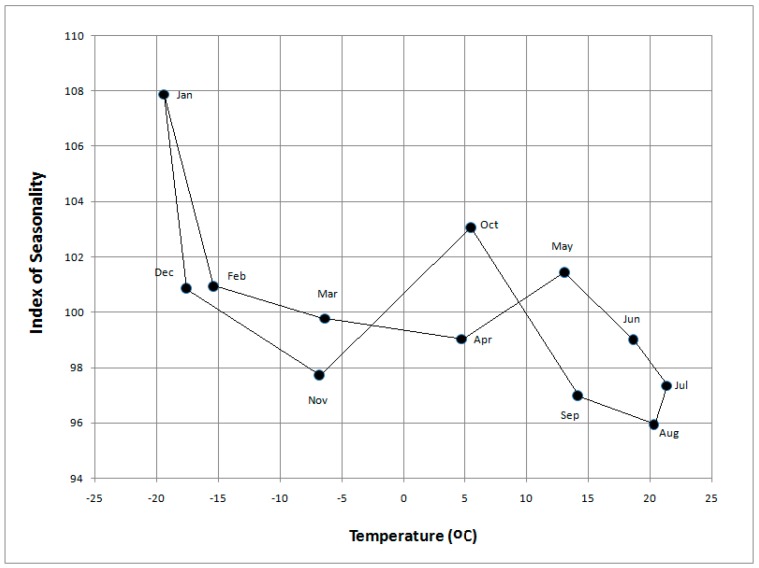
Monthly Index of Seasonality (IS) for Khabarovsk over the period 2000–2012 based on mean daily all-cause mortality for all age groups *versus* mean monthly temperature.

A plot of mean monthly air temperature *vs.* mean monthly mortality for the study period for all age and gender groups exhibits some interesting results (Figure 3). Mortality peaks in January when it is about 13% higher than the summer minimum in August. The overall pattern shows a diminished impact on mortality by the end of summer when it is likely that acclimatization to heat is fully acquired. Most interesting is the response shown in the transition that follows as cooler conditions arrive in autumn (Figure 3), with a pronounced rise in mortality during October, especially for those over 65 years of age. The equivalent rise during the transition from winter to summer shows up late in spring, in May. It is noteworthy that mortality in the acclimatization transition from summer to winter is higher than in the transition from winter to summer. If the pattern was one of a straight forward temporally-autocorrelated relationship, the transitions between winter and summer would be approximately linear and identical.

### ATSI as an Indicator of Mortality

**Figure 4:**
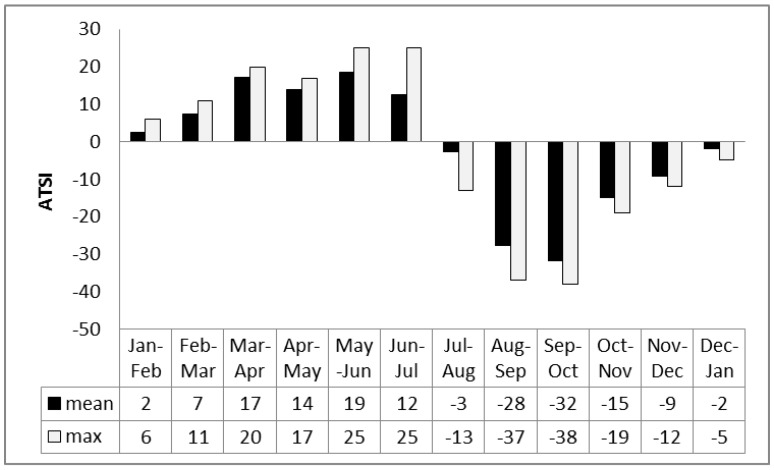
Differences in ATSI between consecutive months for Khabarovsk (2000–2012).

We focus now specifically on the effects of the acclimatization process expressed by ATSI, which is the physiological strain caused by a change in respiratory heat loss between consecutive months, using Equation (5). Results show minimum ATSI values both for winter and summer months, a time when respiratory heat loss is similar for neighboring months (Figure 4). Maximum values are characteristic of the shoulder seasons. The thermal load on respiration is higher for changeover from summer to winter, especially when at the tending September to October transition (Figure 4). This is further evidence of a link between increased mortality and acclimatization strain.

**Figure 5:**
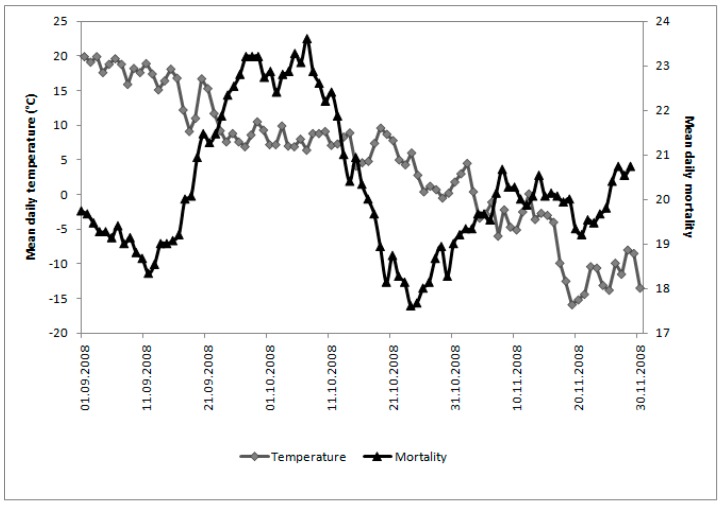
Mean daily temperature and 15-day running mean of daily mortality for Khabarovsk over the autumn of 2008 (1 September to 30 November).

A 13-year period is too short to claim that it captures seasonal transitions that are typical. However, because the autumnal transitions for each year are similar (Table 1), the year 2008 is used to examine this in more detail. Figure 5 gives some idea of seasonal decrease in mean daily temperature. From 1 September to 30 November, air temperature steadily drops from +19.9 to −13.5 °C, with short periods of warm and cool spells and the lowest temperature −15.9 °C on 19 November. Figure 5 also shows changes in the 15-day running mean of daily mortality for the same period, with a pronounced peak value at the end of September through the first 10 days of October. This peak is 25% higher than one month earlier during the first 10 days of September.

**Table 1:** Mean monthly air temperature and standard deviation (°C) for autumn (September to November) and the preceding month for Khabarovsk, 2000–2012.

Year/Month	August	September	October	November
2000	21.7 (±2.51)	14.9 (±3.77)	3.8 (±3.67)	−9.4 (±7.27)
2001	20.5 (±3.06)	12.6 (±4.31)	5.9 (±4.10)	−5.6 (±6.26)
2002	18.3 (±3.18)	14.1 (±3.51)	4.3 (±4.73)	−10.4 (±4.44)
2003	18.0 (±2.25)	13.4 (±2.70)	5.2 (±3.08)	−8.0 (±5.64)
2004	19.3 (±2.11)	15.0 (±2.81)	5.5 (±4.82)	−3.4 (±4.87)
2005	20.8 (±3.80)	15.2 (±3.08)	5.7 (±5.01)	−5.5 (±5.96)
2006	22.1 (±2.22)	14.3 (±3.04)	5.2 (±5.85)	−7.6 (±5.99)
2007	20.7 (±2.73)	14.6 (±3.19)	5.8 (±4.95)	−6.5 (±6.78)
2008	20.1 (±2.27)	14.4 (±4.47)	5.8 (±3.09)	−6.7 (±5.66)
2009	19.8 (±4.28)	13.6 (±3.08)	5.9 (±4.37)	−8.6 (±7.05)
2010	20.8 (±2.06)	14.5 (±5.39)	5.5 (±5.03)	−4.7 (±5.26)
2011	20.9 (±3.11)	12.5 (±3.86)	6.9 (±4.09)	−5.8 (±6.83)
2012	20.0 (±2.66)	14.5 (±4.01)	5.9 (±3.99)	−6.8 (±5.88)
Mean	20.2 (±2.79)	14.1 (±3.71)	5.5 (±4.37)	−6.8 (±5.99)

**Figure 6:**
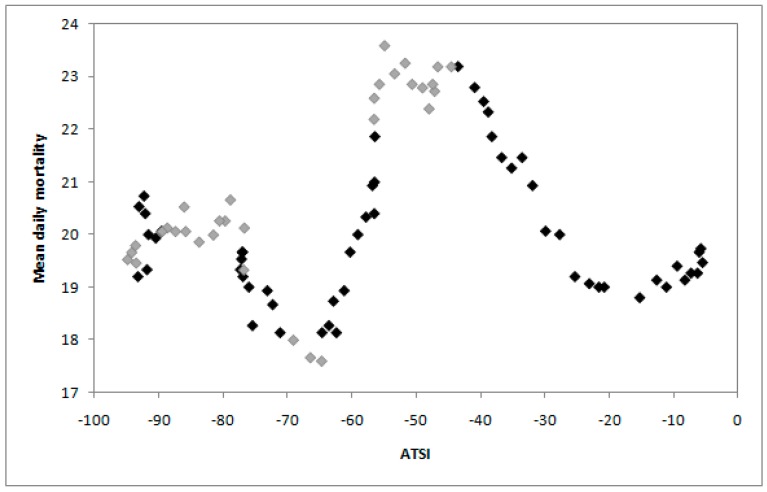
Plots of 15-day running means of daily mortality *versus* daily ATSI over the autumn of 2008 in Khabarovsk. Darker data points indicate Pearson correlation coefficients (*r*) between ATSI and mortality higher than ±0.63; lighter data points indicate *r* values lower than ±0.63.

**Figure 7:**
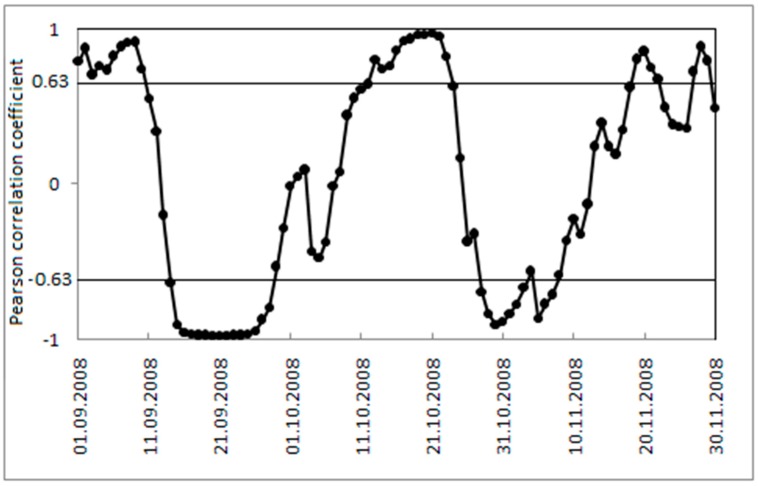
The Pearson correlation coefficient *r* between daily ATSI and 15 day running mean of daily mortality in 10-day blocks for Khabarovsk over the autumn of 2008 (1 September to 30 November). Values ±0.63 indicate critical values of the Pearson correlation coefficient for 0.05 level of significance.

A plot of 15-day running mean of daily mortality in Khabarovsk *versus* daily ATSI, calculated using Equation (6), over the autumn of 2008 is given in Figure 6. There are several short periods through the season with a strong physiological response of the human body to changes in the physical environment expressed in mortality dependence on changes in respiratory heat loss. To examine this relationship further we use the Pearson correlation coefficient in blocks of 10, 15, 20 and 30 days. The strongest correlation is for a short interval of 10 days that is why we give the results for this period (Figure 7). 

Figure 7 shows correlation between daily ATSI and 15-day running means of daily mortality in 10-day blocks for Khabarovsk over autumn of 2008 for the period 1 September to 30 November with no lag. The critical values of the Pearson correlation coefficient *r* for levels of significance 0.05 and eight degrees of freedom (the sample size 10) is 0.6313. The darker data points in Figure 6 and horizontal line in Figure 7 show times during autumn with the strongest relationship with *r* that is higher than critical value 0.6313.

## 4. Discussion

The results of this study confirm that overall seasonal mortality patterns are similar to those found elsewhere in the Northern Hemisphere, including Europe, Asia and the USA [49,50,51,52], where mortality is highest in the winter and lowest in the summer. By August, mortality reaches its lowest point in the annual cycle, when, according to [13], the impacts of heat tend to become diminished. This may be due to mortality displacement or acclimatization. Here we show that mortality rises in the transitional seasons. It is most noticeable in October but there is a secondary jump in May (Figure 1 and Figure 3). Very similar plots for mortality distribution in the USA through the transition seasons have been identified by Davis and Nelson [19] and by Kalkstein [50] for the U.S. metropolitan statistical areas of Minneapolis and Detroit with a two-step rise in mortality.

When mortality is considered in terms of the acclimatization process during the transition seasons of autumn and spring (Figure 1, Figure 2, Figure 3 and Figure 4), sensitivity to a transition to cold is greater than sensitivity to the transition to heat. In other words, acclimatizing from warm conditions to cold appears to exert more strain on the human body than acclimatizing from cold to warm conditions. For this reason we focus on the autumn season (Figure 5, Figure 6 and Figure 7). The results are in accord with our earlier research that suggests the physiological sensitivity per unit ATSI is greater in warm-to-cold acclimatization than it is in the cold to warm transition [42]. This interpretation is supported by known physiological strain mechanisms and the results of laboratory experiments reported in the literature. These are briefly reviewed below.

Several studies have examined a range of mechanisms linking ambient air temperature decrease and adverse effects on lung function. Almost 100 years ago Swift [53] showed that low temperature shivering is accompanied by hyperventilation, which may also increase heat loss via the respiratory system mainly in the form of the heat of vaporization from moisture flux from the lungs. Much more recently, Li *et al.* [54] showed that a decrease of temperature is associated with a reduction in peak expiratory flow, and that a decrease in lung function is related to mortality and morbidity from chronic illnesses such as cardiovascular and respiratory disease. Low temperatures have several effects. They may induce bronchoconstriction and the physiological consequences associated with it [55]. They may also cause airway inflammatory changes through cytokine activation [56]. In addition, they are associated with increases in peripheral vasoconstriction, effects on the body’s haemostatic system [8] and a reduction in lung capacity [57]. In experiments by Driessen *et al*. [58], lung function decreased with airway cooling both in healthy and asthmatic adults as well as children. In addition, exposure to low temperatures is associated with changes in blood pressure, an increase in blood viscosity, levels of red blood cell count and peripheral vasoconstriction by which the lung function is influenced [59].

The work reported here deals with data from one geographic area, thus the results are limited by this regional focus. Nevertheless, the choice of the particular study site allows for assessment of the bioclimatic risks involved in transitional periods of the year for a region characterized by seasonal climatic extremes, as well as an opportunity to test the performance of ATSI with empirical data. The next step might be research using larger datasets and a range of types of mortality or morbidity data to examine relationships in more detail.

Future research might also address the possibility of using respiratory health data specifically in place of all-cause non-accidental mortality data to identify the adaptive consequences of seasonality. However, as discussed earlier, thermal environmental conditions can have a deadly effect on the health of individuals who are already ill from other causes, and frequently the other causes are given as the reason for death. Moreover, thermal conditions may affect individuals differently depending on the disease. Because they are difficult to diagnose with certainty, these deaths can be misclassified as due to causes other than respiratory. Even for healthy individuals, meteorotropic reactions by the human body due to a lack of acclimatization to heat or cold centers on respiratory system, as discussed by [42]. The respiratory organs cannot be protected because humans can do nothing to prevent heat exchange resulting from the ambient air entering into the body’s core area, the lungs, through the respiratory tract. There are other considerations.

The process of acclimatization to heat and cold through respiration is an adjustment process that involves both the lining of the airway and ventilation rate of the respiratory system. The extent of aerobic supply to the body in high latitudes shows distinct seasonal changes [42]. In changing to cold conditions, the breathing rate or volume of air inhaled—or both—with each breath is decreased involuntarily because of bronchial constriction in the upper airway due to cooling and drying stimuli. A consequence of this is that oxygen supply to the lungs is reduced. Moreover, the body is required to produce more energy in cold conditions, which requires a greater supply of oxygen. The resulting discrepancy adds to the physiological strain on the body. The process requires time for lung tissue to adjust so that oxygen supply is redressed in the cold conditions to which the body is newly exposed [42].

## 5. Conclusions

Human mortality is closely related to natural climate-determined levels of thermal environmental stress and resulting thermophysiological strain, among other things. To date, most climate-mortality research has focused on seasonal extremes during summer and winter when mortality is highest. Relatively little research has considered patterns of mortality during the transitional seasons when the body is readjusting through acclimatization. To better understand the influences of weather on mortality through the acclimatization process, the aim here is to examine the periods that link summer and winter using ATSI. ATSI centers on heat exchange with the body’s core via the respiratory system, which unlike the body surface cannot be protected. Unlike widely used bioclimatic indices, ATSI is a relative measure, based on physiological responses known to negatively impact on human wellbeing.

The results show that, although mortality peaks in winter (January) and is at its lowest in summer (August), there is not a smooth rise through autumn or a smooth decline through spring. A secondary peak occurs in autumn (October) with a smaller jump in May. This suggests the acclimatization transition from warm-to-cold produces more thermophysiological strain than the transition from cold-to-warm, which is apparent in the high coefficient of correlation between mortality and ATSI in autumn. The results show the extent to which biophysical adaptation plays a role in increased strain on the body during re-acclimatization and for this reason is a more appropriate climatic indictor than air temperature alone.

The Index of Seasonality (IS) stands out as a useful metric for quantifying “seasonality”. IS captures a noticeable rise in mortality associated with acclimatization strain, especially from the hot to cold transition in autumn shown by a distinct October spike in deaths, in particular, those over 65 years of age. Therefore, the results confirm that the most severe thermal strain occurs with the adjustment shift from hot-humid to cold in that mortality data clearly show the sensitivity of the body to the acclimatization process to cold during autumn is greater than the seasonal shift to heat during spring. However, this may apply for what people experience in the very large shifts in seasonal weather conditions such as in the Russian Far East and may not be the case in other climatic regimes.

The results provide an assessment of the bioclimatic risks involved in transitional seasons for a region characterized by seasonal climatic extremes, as well as an opportunity to test the performance of ATSI with empirical data. Future research using spatially different datasets and a range of types of mortality or morbidity data could provide more detailed results. This information could be handy in planning and managing health services to the public and measures that might be used to help mitigate impacts.
